# Load Management After Gluteal Tendon Repair: A Controlled Laboratory Study

**DOI:** 10.1002/jor.70107

**Published:** 2025-12-13

**Authors:** Alexander Derksen, Celina Wicke, Eike Jakubowitz, Stefan Budde, Christof Hurschler, Henning Windhagen, Michael Schwarze

**Affiliations:** ^1^ Hannover Medical School, Department of Orthopaedic Surgery DIAKOVERE Annastift Hannover Germany; ^2^ Laboratory for Biomechanics and Biomaterials, Department of Orthopaedic Surgery DIAKOVERE Annastift Hannover Germany; ^3^ Evangelisches Klinikum Bethel Universitätsklinikum OWL der Universität Bielefeld Bielefeld Germany; ^4^ Department for Medical Technology Hochschule Bremerhaven Bremerhaven Germany

**Keywords:** gait analysis, gluteal tendon repair, gluteus force, gluteus medius, GTPS, rehabilitation after gluteal tendon repair

## Abstract

Tendon lesions of the gluteus medius muscle require surgical repair if conservative treatment fails. Postoperatively, the success of the therapy is influenced by the loading conditions during follow‐up treatment. Excessive loading of the hip joint during walking after surgery – and consequently, potential overload of the repaired gluteal tendon – could jeopardize the success of the therapy. In this controlled laboratory study, six subjects performed an instrumented 3D gait analysis using optoelectronic motion capture under three different loading conditions: full weight‐bearing, partial weight‐bearing, and full unloading with crutches. Gait data were used *in silico* in an inverse dynamics multi‐body simulation to calculate tendon loading. Peak loads occurred on the gluteal tendons and the hip joint during the stance phase. The force on the gluteus medius tendon during full weight‐bearing amounted to *F*_*me*_ = 12.0 ( ± 1.5) N/kg BW, while the force on the gluteus minimus tendon was *F*_*mi*_ = 5.0 ( ± 1.6) N/kg BW. Partial weight‐bearing reduced these loads by ≈ 68%, whereas full unloading led to a ≈ 88% reduction compared to full weight‐bearing. In the hip joint, the average maximum load during full weight‐bearing reached *F*_*H*_ = 39.8 ( ± 6.7) N/kg BW. To prevent muscle atrophy after gluteal tendon repair, the highest possible load should be applied without compromising suture integrity. Full unloading should be implemented if the tendon and/or bone integrity is compromised and suture stability is reduced. However, if stability is sufficient, partial weight‐bearing is recommended.

## 1 Introduction

The function of the gluteus medius muscle plays a crucial role in stabilizing the pelvis during gait [1]. In the case of complete functional failure, gait becomes unsteady, leading to a limp, which may cause affected individuals to rely on assistive devices [1].

Gluteal tendon pathologies, such as tendinopathies and tendon lesions of the gluteus medius muscle, are the most common cause of lateral hip pain [1, 2]. The prevalence increases in females and with advancing age [1, 3]. Tendon lesions can have either a traumatic or a degenerative cause [1]. In the case of a degenerative cause, tendinopathy (inflammation without macroscopic tearing) is the underlying factor [2]. The pathomechanics of gluteal tendinopathy are complex and multifactorial [3, 4]. One possible mechanical cause is assumed to be the compression of the tendons under the iliotibial tract with simultaneous tensile stress [5]. This occurs primarily during hip adduction when the iliotibial tract is passively pre‐tensioned and additionally actively tensioned by the abductors [5]. Other predisposing factors include a decreased femoral neck angle (coxa vara), which increases compression, and a relative weakness of the hip abductors in relation to the iliotibial tract tensioners [5].

While conservative measures, such as physiotherapeutic training, weight reduction, and injections are indicated in the early stages, higher‐grade or therapy‐refractory lesions require surgical treatment [1, 2, 4].

The primary goal of a repair surgery is a stable fixation of the ruptured tendon to the greater trochanter to enable healing and to avoid re‐tears during the early rehabilitation phase, especially due to overstressing of the resulting construct (tendon, suture material, and adjacent bone) [1]. Such surgical repair procedures include different suturing techniques, such as direct tendon suturing, tendon transfer, or tendon augmentation grafts [1]. In particular, the suture configuration and the strength of the suture construct, which consists of the tendon, bone, and suture material, differ. The choice of procedure depends on the extent of tendon degeneration and thus the quality of the remaining tendon tissue, as well as the size of the defect [1]. Prerequisites for successful reconstruction are intact innervation and a low degree of fatty degeneration of the gluteal muscles [1]. Failure of the repaired gluteus medius tendon occurs in up to 35.7% of cases, depending on the knotting technique and bone preparation [6].

In addition to primary stability, an appropriate postoperative rehabilitation phase is crucial for long‐term therapeutic success. Ensuring adequate tendon healing requires a balance between avoiding overloading of the suture while maintaining a progressive increase in loading to maintain muscle and tendon function and therefore avoid severe muscle atrophy.

To date, the loads imposed on a gluteal tendon repair during early postoperative, crutch‐assisted gait particularly under graded weight‐bearing have not been quantified. Prior studies pursued different aims and yielded insights specific to gluteal muscle mechanics under unassisted conditions. In dysplastic hips, subject‐specific models showed shortened abductor (gluteus medius) moment arms, more medially directed lines of action, higher abductor forces, and elevated medial/resultant hip joint reaction forces during level walking [7]. Estimates of hip joint reaction and muscle forces further depended on model geometric specificity (generic vs. subject‐specific) during gait [8]. During deep squat, psoas and semimembranosus forces were higher in asymptomatic cam and control cohorts than in patients with FAI; the ACM group also showed higher gluteus medius force than controls, while FAI demonstrated reduced posterior/superior/total hip contact forces [9]. None of these studies quantified tensile loads at the gluteus medius/minimus tendon insertions or investigated crutch‐assisted, graded loading (full, partial, non‐weight‐bearing) relevant to early postoperative protection after gluteal tendon repair.

Deriving the load requirements for the primary stability of such a tendon suture as well as recommendations for a safe early postoperative rehabilitation, requires an investigation of the forces acting on the gluteal tendons under typical clinical loading conditions.

Therefore, the aim of this study was to quantify these forces on the gluteus medius and minimus muscle tendons under different loading conditions during gait with and without forearm crutches (full weight‐bearing, partial weight‐bearing with crutches, and full unloading with crutches). The results are intended to contribute to deriving recommendations for safe and effective early rehabilitation after gluteal tendon repair. A better understanding of the load requirements on the suture construct and pathomechanical relationships should eventually lead to improved quality of care and reduced complication and recurrence rates.

We hypothesized that gluteal tendon loading decreases progressively from full to partial weight‐bearing and is minimal during full unloading, thereby reducing the risk of suture failure.

## 2 Methods

This controlled laboratory study was approved by the Ethics Committee of Hannover Medical School (No. 10269_BO_K_2022) and conducted in compliance with the Declaration of Helsinki. Kinematic and kinetic data from three‐dimensional gait analysis were used *in silico* in a multi‐body simulation to calculate muscle and tendon loading via inverse dynamics. During this process, particular consideration was given to the external forces and moments acting on the tendons of the gluteus medius and minimus muscles. Three‐dimensional gait analysis allows the interaction between muscle activity and joint movement across a body segment or even the entire body during different activities to be digitally simulated and evaluated [10]. This provides the opportunity to virtually analyze the load requirements on the musculoskeletal system, such as the force transmission from individual muscles to their tendons. This study used three‐dimensional gait analysis to precisely determine muscle/tendon loading, taking into account external forces and moments acting on the human body and, specifically, in the tendons of the gluteus medius and minimus muscles.

### 2.1 Participants/Samples

A total of six healthy participants (S1–S6) were enrolled (mean age: 25.5 ± 3.4 years; body height: 1.74 ± 0.05 m; body weight: 73.1 ± 16.4 kg; three female). Participants were required to be between 18 and 35 years old. Individuals with restricted lower limb mobility, neurological disorders, or cognitive impairments were excluded. Before participation, all subjects provided written informed consent.

### 2.2 Gait Analysis

Three‐dimensional joint kinematics of the lower extremities during barefoot walking on a 10 m walkway were captured using an optical infrared motion capture system. The system consisted of twelve MX cameras (Vicon Motion Systems Ltd., Oxford, UK) controlled via Nexus software (Version 1.8.5) and operated at a sampling rate of 200 Hz. Ground reaction forces were measured using two force platforms (Type BP400600, AMTI, Watertown, USA) at a sampling rate of 1 kHz. Participants were equipped with the Helen Hayes lower‐body reflective marker set [11].

All participants performed six valid barefoot gait trials per weight‐bearing condition at a self‐selected walking speed, ensuring valid foot strikes on the force platforms. Participants used their natural gait pattern during the full weight‐bearing condition. For partial weight‐bearing, participants used bilateral forearm crutches and loaded the tested leg with 150–200 N; this target was practiced and verified on the two force plates. This target corresponds to the commonly prescribed 15–20 kg during early postoperative care after gluteal tendon repair [12]. Given the variability of published aftercare protocols in both loading intensity and duration [6, 12, 13, 14, 15, 16], we opted for an absolute, force‐based target that can be monitored on force plates and aligns with our institution's postoperative care plan.

Full unloading was performed with bilateral forearm crutches; the instrumented (right) foot was allowed to touch the second force plate for balance but remained unloaded (vertical GRF ≈ 0 N). Kinematic data were processed using Nexus software and smoothed via spline interpolation with a Woltring filter. Joint kinetics of the leg were computed using inverse dynamics.

### 2.3 Multi‐Body Simulation

Kinematic and kinetic gait data were then used in a multi‐body simulation program (AnyBody Modeling System, AnyBody Technology A/S, Aalborg, Denmark). The program was extended with the Simple Lower Extremity Model from the AnyBody Managed Model Repository (AMMR V2.4.4). The simulation model allowed for dividing the gluteus medius muscle into two functional segments: the anterior and the posterior gluteus medius. Three functional segments were considered for the gluteus minimus muscle: the anterior, medial, and posterior gluteus minimus. As a result, the model provided direct force data from muscles and thus allowed for a derivation of respective gluteus medius and the gluteus minimus muscle tendon forces *F*_*me*_ and *F*_*mi*_. They were normalized to the body weight (BW) and expressed in Newton per kilogram of body weight (N/kg BW).

**Figure 1:**
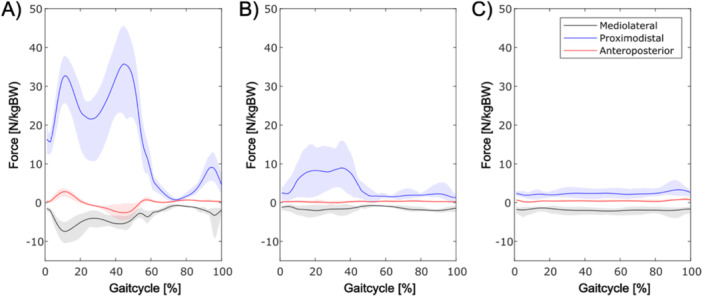
Overview of the hip joint force profiles in three directions (mediolateral, proximodistal, and anteroposterior) during a gait cycle. (A) full weight‐bearing, (B) partial weight‐bearing, and (C) full unloading. The lines represent the mean values of the acting forces, and the shaded areas indicate the standard deviation. The x‐axis corresponds to the gait cycle in percent, and the y‐axis represents the normalized force N/kg body weight (N/kg BW). The highest forces occur in the proximodistal (z) direction.

To verify the physiological plausibility of the simulated hip contact force during level walking, stance‐phase waveforms were compared against the instrumented hip data set reported by Bergmann et al. [17]. Data were time‐normalized to 0%–100% stance; we compared peak magnitude and peak timing with the benchmark mean curve descriptively. This descriptive comparison was meant for rough corroboration of the joint loading and was not intended as a formal validation of individual muscle forces. An overview of the resulting hip joint force profiles over the gait cycle is shown in Figure 1.

Abbreviations used: GMED = gluteus medius, GMIN = gluteus minimus; Fme, Fmi denote tendon‐transmitted forces normalized to body weight (N/kg BW).

### 2.4 Analysis and Evaluation

The resulting simulation model forces on the gluteal muscles (gluteus medius and minimus) were compared between the three different weight‐bearing conditions using a custom‐made MATLAB coding (R2021a, MathWorks Inc., Natick, MA, USA). Analyses were not hypothesis driven and therefore not supported by formal statistical tests. Force data were time‐normalized to 0%–100% gait cycle and averaged. We report mean ± SD of peak forces (N/kg BW) per muscle (and segment) and condition; minima and maxima denote extreme values.

## 3 Results

**Figure 2:**
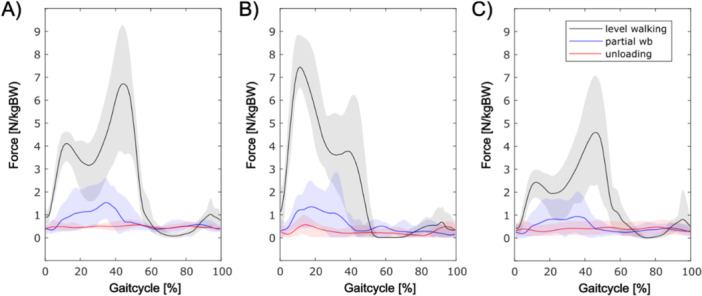
Muscle force comparison between gluteal muscle parts during different loading conditions (A = anterior gluteus medius, B = posterior gluteus medius, C = gluteus minimus).

A maximum force averaged from single parts of the gluteus medius muscle (Figure 2) of *F*_*me*_ 12.0 (±1.5) N/kg BW was shown acting on its tendon during full weight‐bearing. This loading can be reduced by ≈ 68% to *F*_*me*_ 3.9 (±1.6) N/kg BW through partial weight‐bearing (defined as 150–200 N vertical ground‐reaction force, measured at the force plate) and by 88% to *F*_*me*_ 1.3 (±0.3) N/kg BW through full unloading. For the gluteus minimus muscle, a mean maximum force of *F*_*mi*_ 5.0 (±1.6) N/kg BW is acted on its tendon during full weight‐bearing. This can also be reduced by 72% to *F*_*mi*_ 1.4 (±0.7) N/kg BW through partial weight‐bearing and by 86% to *F*_*mi*_ 0.7 (±0.1) N/kg BW with full unloading (Table 1).

**Table 1:** Forces generated by the two gluteal muscles during the three loading scenarios (N/kg BW; SD = standard deviation).

Muscle	Force	Full weight‐bearing (N/kg BW)	Partial weight‐bearing^a^ (N/kg BW)	Full unloading^a^, (N/kg BW)
Gluteus med. (GMED)	Mean (SD)	12.0 (1.5)	3.9 (1.6)	1.3 (0.3)
	Max	15.3	7.7	2.4
	Min	9.4	1.0	0.6
Gluteus min. (GMIN)	Mean (SD)	5.0 (1.6)	1.4 (0.7)	0.7 (0.1)
	Max	7.1	2.7	1.0
	Min	3.1	0.5	0.4

^a^ Partial weight‐bearing: target vertical GRF 150–200 N (force plate verified), Full unloading: vertical GRF ≈ 0 N (toe‐touch balance only).

During the stance phase with full weight‐bearing, an averaged maximum force acting on the hip joint was calculated with *F*_*H*_ = 39.8 (±6.7) N/kg BW (Figure 1). Thus, the magnitude and timing of forces exhibited strong qualitative coherence with the reference values reported by Bergmann et al [17]. It was reduced to *F*_*H*_ = 12.7 (±5.7) N/kg BW during partial weight‐bearing and to *F*_*H*_ = 4.9 (±1.4) N/kg BW during full unloading. This corresponds to a reduction of ≈ 68% under partial weight‐bearing and ≈ 88% under unloading relative to full weight‐bearing.

**Figure 3:**
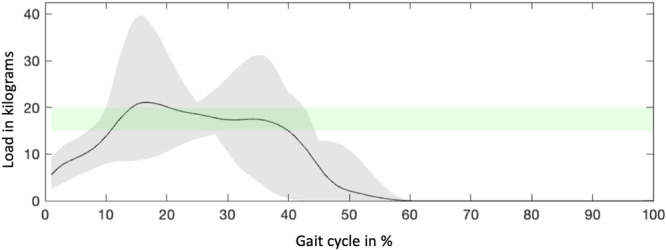
Mean ground reaction forces (GRF) on the right leg during walking with forearm crutches under prescribed partial weight‐bearing.

The average partial weight‐bearing of around 200 N (150 – 200 N) was maintained but with substantial variability among subjects (Figure 3).

## 4 Discussion

The results of the present study show a clear decrease in tendon loading through a partial weight‐bearing or even full unloading of the affected leg compared to full weight‐bearing during postoperative mobilization into gait on crutches.

This is particularly necessary for early rehabilitation after gluteal tendon repair to enable safe healing of the tendon into the bone. Therefore some aspects of previously described aftercare protocols as well as biomechanical stability studies of tendon repair are discussed in the context of the present loading estimates to outline potential implications for early rehabilitation, no direct comparison between clinical aftercare protocols and tendon–suture complex stability was undertaken. Comparing the three loading conditions, tendon loading decreased by ≈68% (GMED; ≈72% for GMIN) during partial weight‐bearing relative to full weight‐bearing. The difference between partial weight‐bearing and full unloading is comparatively small (decrease of 2.6 N/kg BW). However, full unloading may be necessary under certain conditions, such as a markedly reduced stability of the tendon‐suture construct.

The loading instructions specified in clinical studies on the topic of gluteal tendon repair with indications for postoperative aftercare protocols were reviewed. These agree on recommending reduced loading in the stance phase to avoid overloading the tendon‐suture construct. However, the intensity varies. While some authors had their study population perform partial weight‐bearing, full unloading was prescribed in other studies [6, 13, 14, 15, 16]. At the same time, the information on the time periods for reduced loading varied. Thaunat et al. recommended avoiding active exercise for the first 6 weeks. Only flexion up to 90° was allowed. All other leg movements and leg loading during locomotion should be avoided [15]. In the authors' view, stiffening of the hip joint due to inactivity is very rare, so restrictive aftercare to reduce the risk of an inflammatory reaction of the tendon during healing is sensible [15]. Full unloading was also recommended by Kocaoglu et al. in their 3‐phase protocol of postoperative aftercare, while passive exercise of the hip joint was allowed in all directions of movement [6]. Saltzman et al. also described a standardized 3‐phase protocol of postoperative aftercare, with partial weight‐bearing allowed while walking with a walker or crutches [16]. Only Davies et al. described a precise specific value with a load of 25% of body weight [13]. However, this information varies in tendon loading depending on individual body weight, so heavy patients have a higher risk of overload. No study was found in which aftercare was allowed under full weight‐bearing, as there is most likely agreement that the repaired tendon is overloaded in this case.

If the load on the tendon of the gluteus medius muscle is considered in isolation, this study shows that there is an average maximum load of *F*_*me*_ = 3.9 N/kg BW with a partial weight‐bearing of 200 N. At the same time, the tendon of the gluteus minimus muscle is loaded with *F*_*mi*_ = 1.4 N/kg BW. With full unloading, the forces can be more than halved to *F*_*me*_ = 1.3 N/kg BW and to *F*_*mi*_ = 0.7 N/kg BW compared to partial loading. This results in a load requirement on the suture construct of the gluteus medius tendon of *F*_*me*_ = 351 N during partial weight‐bearing in the early postoperative phase, taking into account a body weight of 90 kg.

Biomechanical studies with repair techniques of the gluteal tendons vary widely in their results (454–152 N). The study by Flynn et al. shows that the double‐row knotting technique achieves an ultimate load from *F*_*ul*_ = 152.1 (±68.6) N to *F*_*ul*_ = 161.1 (±72.0) N, depending on whether an open or endoscopic surgical technique was used [18]. Considering the results from our study, the values are well below the stability requirements for partial loading in a person with a BW of 90 kg. Further studies show ultimate loads of up to *F*_*ul*_ = 454 N but with a relatively large standard deviation. Twardy et al. compared the double‐row technique against the classic Mason‐Allen technique in single‐row and concluded that the DR technique provides significantly higher stability with *F*_*ul*_ = 339.1 (±144.4) N compared to *F*_*ul*_ = 209.6 (±62.1) N [19]. In another biomechanical stability study on human cadavers, Kahlenberg et al. compared double‐row and single‐row techniques [20]. They also concluded that the single‐row technique was significantly weaker (*F*_*ul*_ = 348.0 ± 250.9 N compared to *F*_*ul*_ = 188.3 ± 36.8 N), although the limitation of high standard deviation also exists here. Dishkin‐Paset et al. were slightly higher in the results of their study and observed a stability of *F*_*ul*_ = 439 (±127) N compared to *F*_*ul*_ = 454 (±179) N between two different DR techniques on human cadavers [21].

Three of the four studies show that the double‐row knotting technique exceeds the average stability requirement for the example patient mentioned, but the standard deviation is always high. Thus, safe stability cannot be assumed with the DR technique considering an average aftercare male patient with a body weight of 90 kg, resulting in *F*_*me*_ = 351 N. Furthermore, considering the requirements, the single‐row technique should neither be used nor treated with partial weight‐bearing to avoid the high risk of re‐tear. Another aspect that should be considered is the anchoring of the suture construct in the bone. The stability varies depending on the bone preparation, as described by Putnam et al. The stability of the suture anchors achieves a load of 206.7 (±75.0) N for non‐decorticated bone surface compared to decorticated bone ± 152.3 (±60.2) per anchor. The anchoring stability increases with the number of suture anchors. With the usual 3–4 anchors for a DR technique, sufficient suture stability can be assumed for an exemplary patient of 90 kg BW.

The present loading estimates across full weight‐bearing, partial weight‐bearing, and unloading provide an overview of tendon loading of the gluteus medius and minimus and may support individualized dosing of early postoperative loading to avoid overload. These results can serve as a basis for developing targeted training and rehabilitation strategies tailored to the demands of the repaired gluteal tendons.

In future studies, wearables could be used to objectively measure adherence to prescribed loading targets (e.g., 150–200 N) in early postoperative patients outside the gait laboratory. Assessments should also go beyond steady‐state walking on forearm crutches to include everyday tasks and ranges of motion not required for walking (e.g., stairs, sit‐to‐stand, sitting, turning, inclined walking). Subject‐specific and EMG‐informed models should be included so that loading can be linked to repair‐construct mechanics to define thresholds and progression; and the benefit of wearable feedback on adherence and outcomes should be tested as a useful extension of the rehabilitation strategy, with the aim of reducing risk to the recently repaired tendon.

### 4.1 Limitations

This study does not make a direct comparison between clinical aftercare protocols and tendon–suture complex stability. Prior protocols and biomechanical stability studies are considered only to contextualize the present loading estimates. Activities beyond steady‐state level walking (e.g., stair negotiation, sit‐to‐stand, sitting, turning, inclined walking) were not assessed, as the objective was to characterize loading during level gait. Partial weight‐bearing was imposed in a controlled laboratory setting with healthy participants; generalizability to postoperative patients and real‐world crutch patterns may be different. This study lacks individual anatomical variations of the femur, such as coxa vara or coxa valga, as well as torsional changes, which can alter the biomechanical force ratios. Different variations of the pelvis can also influence muscle activity and tendon loading, which was not considered in this study. The small sample (*n* = 6) further limits statistical inference and subgroup analyses.

## 5 Conclusion

In summary, this study shows that partial loading and full unloading of the leg during the stance phase of gait on crutches leads to a clear reduction in the tensile forces of the gluteal tendons (gluteus medius and minimus muscles). Depending on the quality of the tendon‐suture construct, this should be considered when choosing postoperative aftercare, and in cases of doubt, full unloading should be maintained initially.

## Author Contributions

Alexander Derksen: Project planning, manuscript writing, supervision of Celina Wicke. Celina Wicke: Test setup elaboration, data processing, drafting and revision of the manuscript. Eike Jakubowitz: Data processing, drafting and revision of the manuscript. Stefan Budde: Project planning, drafting and revision of the manuscript. Christof Hurschler: Project planning, drafting and revision of the manuscript. Henning Windhagen: Project planning, drafting and revision of the manuscript. Michael Schwarze: Test setup elaboration, data processing, supervision of Celina Wicke, descriptive analysis, data processing and visualization, drafting and revision of the manuscript.

## Ethics Statement

This study was approved by the local ethics committee (Approval date: 03/11/2022, Reference number 10269_BO_K_2022).

## Conflicts of Interest

The authors declare no conflicts of interest.

## Data Availability

The datasets used and analyzed during the current study are available from the corresponding author upon reasonable request.
